# Agricultural Transformation, Nutrition Transition and Food Policy in Africa: Preston Curves Reveal New Stylised Facts

**DOI:** 10.1080/00220388.2018.1430768

**Published:** 2018-02-26

**Authors:** William A. Masters, Nathaniel Z. Rosenblum, Robel G. Alemu

**Affiliations:** * Friedman School of Nutrition and Department of Economics, Tufts University, Boston, MA, USA; ** One Acre Fund, Rubengera, Rwanda; † Friedman School of Nutrition Science and Policy, Tufts University, Boston, MA, USA

## Abstract

This paper uses a Preston Curve approach to test for changes over time in agriculture, nutrition and food policy, comparing national averages in Africa and elsewhere at each level of national income per capita from the 1990s to the 2010s. Our statistical tests and data visualisations reveal that, at each level of income, African countries have faster rural population growth, a larger share of workers in agriculture and lower agricultural labour productivity than countries elsewhere, with no significant shift in these patterns from the 1990s to the 2010s. In contrast, there have been structural shifts towards less child stunting everywhere, and towards more adult obesity in high-income countries. The overall pattern of African governments’ food policies and government expenditures have not shifted, however, as they continue price interventions and low investment levels characteristic of low-income countries around the world.

## 1. Introduction and motivation

In the first 15 years of the twenty-first century many African countries have experienced rapid economic growth and poverty reduction, accompanied by big changes in agri-food systems and human nutrition. This paper places Africa’s recent surge in the context of previous transformations in Asia, Latin America, and Africa itself. We use a wide variety of data to test whether and how Africa’s recent changes in agriculture, nutrition and food policy differ from experience elsewhere, and from the patterns experienced within Africa in previous decades. To describe these relationships and test for structural shifts we use non-parametric regression of each agricultural, nutritional or policy variable on national per-capita income, adapting the Preston curve approach that has been widely used in public health research (Bloom & Canning, 2007) to test for stylised facts about national averages at each income level.

The changes in agriculture and food systems that we observe so far in twenty-first century Africa have been broadly consistent with the patterns first described in modern economic terms by Clark (1940), Chenery (1960) and others. From the start of economic growth, workers move from food production to services and industry in ways that could be explained by productivity growth in any sector, combined with limited opportunities for agricultural expansion due to relatively fixed supply of land and water, and relatively fixed demand for food or other farm products. These structural constraints on expansion of farming ensure that increases in agricultural productivity serve to fuel the growth of nonfarm sectors, while failure to improve farming leaves rural people struggling to feed themselves until they can find nonfarm work.

Regional differences in agricultural transformation and rural poverty are closely linked to the speed and timing of population growth, following paths first described by Dovring (1959) and then documented in detail by Tomich, Kilby, and Johnston (1995). In tropical Asia during the late 1940s and 1950s, and then in Africa in the 1960s and 1970s, sudden introduction of public health programmes in rural areas sharply improved maternal and child health, leading to a child-survival baby boom that raised population growth rates for several decades until fertility rates could decline enough to overcome demographic momentum. Given the small initial number of urban and rural nonfarm jobs, even the rapid year-to-year annual rates of economic growth observed in Asia during the 1980s and 1990s and then in Africa during the 2000s were not enough to absorb all the children of farmers. The result has been a continued rise in rural populations and decline in land available per farmer, despite urbanisation and income growth among non-farmers.

This paper documents the timing and pattern of changes from the 1990s to the 2010s, comparing the experience of African countries to other regions, starting with the agricultural transformation described above, then turning to nutrition and food policy. Nutrition transition, a term coined in the early 1990s by Popkin (1993, 1994), refers to systematic differences in body size and dietary intake associated with economic development. The term focuses particularly on the transition from stunting and underweight that have been successfully addressed over many decades as documented by Fogel (2004), Deaton (2007) and others, to the relatively sudden rise in obesity observed since the 1980s. Stunting results from deprivation in infancy and is rarely reversed, while obesity results from later weight gain, so the two forms of malnutrition often coexist in the same people. Shifts in overweight and obesity by region are shown in Stevens et al. (2012), and changes in specific dietary risk factors are linked to their overall burden of disease in Lim et al. (2013).

The agricultural transformation and nutrition transition described above are heavily influenced by government interventions, in ways that alter the path and may sometimes accelerate and sometimes slow the pace of change. Most notably, as documented most recently by Anderson and Nelgen (2013), through the twentieth century agricultural transformation was generally accompanied by the apparent paradox that governments in low-income countries typically intervened to reduce food prices, while middle- and high-income country governments typically intervened to raise them. This food policy transition was seen as paradoxical because, within each country, it tended to redistribute income from the poorer majority to a richer minority, delaying the agricultural transformation towards a permanently smaller share of resources devoted to food production. Policy-makers may have also distorted prices towards staple foods, delaying any dietary transition towards healthier foods. We conclude this review by examining these food policy trends in Africa relative to other regions.

## 2. The demographic context

Changes in agricultural life are driven in part by year-to-year variation in the number of people living in rural areas, especially in poor countries where most rural people are farming. Given a fixed total area of accessible land, water, forests and other natural resources, an increase in the number of rural people reduces the amount of those resources per person, with less land area available per rural household. During economic development, demographic transition towards slower growth of the total population combined with outmigration from rural areas gradually reduces the rate of rural population growth to below zero, at which time land area per rural person can increase over time. The year when rural population growth crosses zero has been called a ‘structural transformation turning point’ (Tomich et al., 1995), after which each remaining rural resident can expand their farm size, contributing to higher rural incomes.

**Figure 1.**
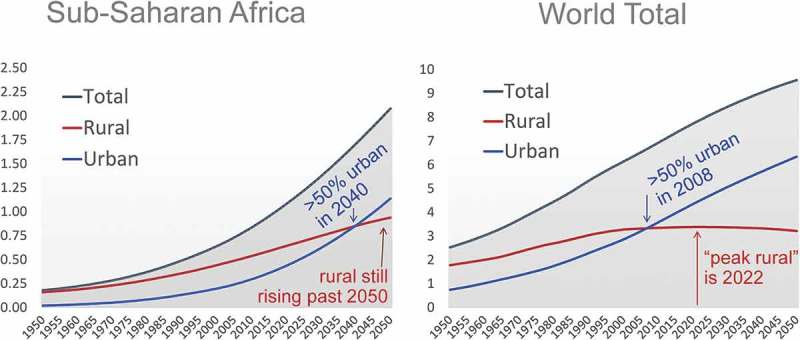
Despite rapid urbanisation, Africa’s rural population will keep growing past 2050. *Notes:* Vertical axis is population, in billions. All data are calculated from United Nations (2014), UN Population Projections and World Urbanization Prospects, 2014 Revision, released July 2014 at http://esa.un.org/unpd/wup.

Figure 1 uses UN population projections and urbanisation prospects from 1950 to 2050 to show how the timing of structural transformation and its turning point differs for sub-Saharan Africa relative to the world as a whole. The top line for each panel is the world or region’s total population, which grows exponentially at an increasing rate in the initial decades of demographic transition when child mortality falls, and continues growing at a declining rate when fertility falls. The dashed line is urban population, which accounts for a small fraction of the total in the early years of structural transformation, and then rises quickly as cities house and employ a rising share of people over time. The change in rural population depends on total population growth minus urban growth, with rural people moving to towns and cities as quickly as migration opportunities allow. The right panel shows how the world as a whole reached 50 per cent urban around 2008, while the combination of slowing total population growth with continued urbanisation puts the turning point towards negative rural population growth around 2022; the left panel shows how population dynamics in Africa follow the same pattern but much later in time with different magnitudes, reaching 50 per cent urban around 2040 and the rural population continuing to grow past 2050.

The results shown in Figure 1 set the stage for our specific results presented below, in which we use Preston curves to show how countries in Africa differ from nations elsewhere.

## 3. Data and methods

To characterise the overall pattern of agricultural change and nutrition transition in Africa and elsewhere from the 1990s into the 2010s, we build on Masters et al. (2016) by constructing Preston Curves that compare the averages observed among countries around each level of national income per capita, measured in real terms controlling for the purchasing power of local currencies obtained from the World Development Indicators dataset of the World Bank. The specific variables we use are described in Table 1, and were chosen to illustrate principal features of three changes: (a) agricultural transformation, described in terms of rural population growth, agriculture’s share of total employment, and the productivity of agricultural workers relative to non-agricultural workers; (b) nutrition transition, in terms of child stunting and adult obesity then diet quality with intake of healthy and unhealthy diets, and (b) food policy in terms of consumer prices and public investment levels.

**Table 1.** Variables used for Preston Curve analyses

Variable		Definition	Source	Years	Countries
National income (independent variable)					
	GDP per capita (PPP, Constant 2011)	National income, adjusted for purchasing power	WB World Development Indicators, 2016	1990–2014	214
Agricultural transformation					
	Rural population growth (pct/yr)	Year-to-year increase in number of people in rural areas	UN World Urbanisation Prospects, 2014	1950–2050	228
	Employment in agriculture (pct of total)	Fraction of all workers who are working in agriculture	ILO World Economic & Social Outlook, 2015	1991–2013	173
	Labour productivity in agriculture relative to other sectors (index)	Value added (VA) per worker in agriculture, divided by VA/wkr in all other sectors	World Bank & ILO, 2016	1991–2013	127
Nutrition transition					
	Stunting prevalence (pct of children aged <5yrs)	Pct of children, based on height for age z scores (HAZ<2)	UNICEF/WHO/World Bank JMP, 2016	1983–2015	146
	Obesity prevalence (pct of adults aged >20yrs	Fraction of adults with body mass index (BMI>30)	Global Burden of Disease project estimates, 2015	1990–2013	188
	Diet quality (score for intake of healthy diets)	Score from 1 to 100, higher = more intake of 10 healthy foods	Average of 10 intake estimates, from Imamura et al., 2015	1990–2010	187
	Diet quality (score for less intake of harmful diets)	Score from 1 to 100, higher = less intake of 7 unhealthy foods	Average of 7 intake estimates, from Imamura et al., 2015	1990–2010	187
Food policy					
	Price policy (proportional increase in consumer prices)	Consumer tax equivalent (CTE) increase in prices due to interventions	Anderson and Nelgin, 2013	1990–2011	82
	Public spending for agriculture and health (pct of gvt budget)	Pct of government spending in health or agriculture	IFPRI SPEED data, 2016	1980–2012	147

*Note*: Each variable is described with citations to data sources where results are presented.

The Preston Curve method allows us to analyse changes over time and differences across countries for all these variables in a consistent manner, testing for statistically significant changes in each region’s means at each level of income between data from before and after 2000. This technique was first developed to identify changes in the relationship between life expectancy and per-capita income (Bloom & Canning, 2007; Preston, 1975), and can be generalised from that to any aspect of socio-economic development. In our tests, if the twenty-first century so far is like the 1990s, in the sense that countries with more purchasing power produce and consumer more of the same things, then countries will have moved along each curve. Any shift in the curves’ level will be due to innovations in global or regional technology, institutions or other structural conditions. The Preston Curve approach provides a powerful test of whether changes are due to innovations that are new to the region as a whole, or can be explained in terms of an established path of socio-economic development associated with per-capita income and purchasing power. Each curve describes the shape and tests for shifts in a bivariate relationship that could then be explained as a function of other variables in other models.

In this paper we test the statistical significance of changes in nine kinds of relationships, each using non-parametric regressions with no assumptions about the shape of development paths. Some variables could be a linear function of national income, but they could also be U-shaped or S-shaped reflecting both consumers’ income elasticities of demand and the political economy of government policies at each level of national income. The significance of changes in that function depends not only on the mean but also variance, which we show with a 95 per cent confidence interval around the estimated mean at each income level in each region. The mean and confidence interval for each variable is estimated at each level of income using a local polynomial regression (Henderson & Parmeter, 2015), implemented in Stata version 14.2 using the command lpolyci. These are Epanechnikov kernel regressions, fitting a polynomial of degree zero so that the estimate at each point is a moving average, imposing a uniform bandwidth set at 0.75 log points of national income to ensure that local means are computed over the same range in each curve for ease of comparison. The result provides both a convenient visualisation and a statistical hypothesis test for the difference in local means at each national income level.

Our approach is statistical but is presented graphically, and for visual clarity all charts are formatted in a consistent manner. Preston Curves with non-overlapping confidence intervals reveal a statistically significant shift from one decade to the next, or significant differences in the pattern between Africa and the rest of the world. African countries are shown using solid squares or circles, and non-African countries are shown with hollow squares or circles. Africa is on the left panel with others on the right where splitting the figure is helpful. If both regions can be shown on a single panel, results for Africa are shown with a solid regression line and others with a dashed line. To compare changes over time, where the data are modelled to fill every year we use data for just 1990 and 2010, and where data are from occasional surveys we use an average of available years in the 1990s and 2010s. The earlier observations are always shown using circles in lighter shades (of green, if colour is available) for the 1990s, and squares in darker shades (of blue) for data for the 2010s.

### 3.1. Agricultural transformation

#### 3.1.1. Rural population growth

**Figure 2.**
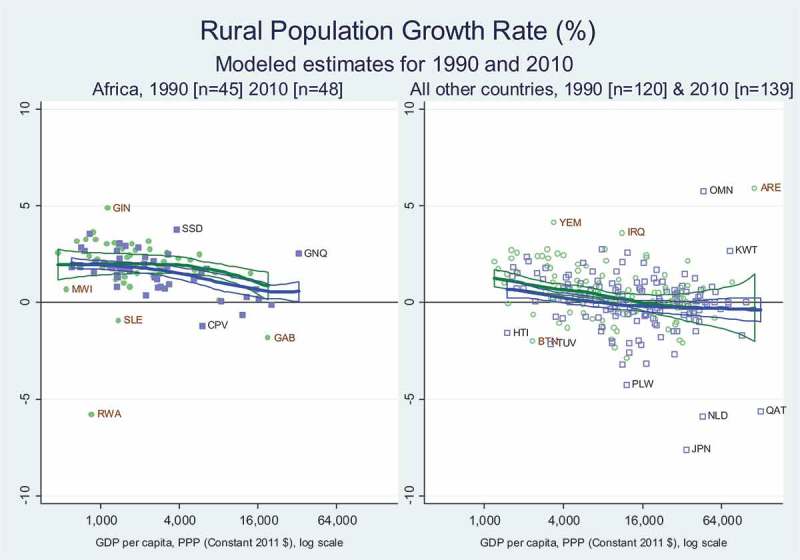
Rural population grew faster in Africa than elsewhere at each income level.

We introduce the Preston Curve method using the same rural population data as Figure 1, presented in Figure 2 in terms of year-to-year growth rates. This and other charts also show the number of observations for each region and year, with notes below the figure and country codes for outliers shown on the chart.

Figure 2 reveals no shift over time within each panel in rural population growth rates at each level of income, but looking across panels there are significantly higher rural growth rates in Africa than elsewhere, at all but the highest levels of income. The gap arises because there is a significant downward income gradient in the rest of the world, from above 2 per cent per year in the poorest places towards zero and then negative rural population growth when income passes about $6000 per year, whereas Africa’s rural population growth rates remain around 2 per cent per year at all but the highest income levels.

The distinctive fact about African demography revealed in Figures 1 and 2 arises due to rapid total population growth and other aspects of demographic change described in Masters et al. (2013). The increases in rural population shown here ensure that, even with very rapid urbanisation and rising nonfarm incomes, the amounts of rural land and water or other natural resources per rural resident is shrinking much faster in Africa than elsewhere, reducing rural income growth.

#### 3.1.2. Employment shares

Another perspective on economic transformation concerns the allocation of labour between agricultural and non-agricultural activity. Yeboah and Jayne (2018) use the limited available time-use data to show how rural households are often engaged in both farm and non-farm work, some of which involves temporary or permanent migration within rural areas as well as to or from urban settlements. Barrett, Reardon, and Webb (2001) show how reallocation over time is driven by the rise of new nonfarm opportunities as well as change in available farmland and other agricultural resources per rural worker. Yeboah and Jayne (2018) find only five African countries with repeated surveys on time use by sector, so for a broader comparison, we use International Labour Organization (ILO) estimates of primary employment in agriculture from the most recent ILO (2015) World Employment and Social Outlook database.

**Figure 3.**
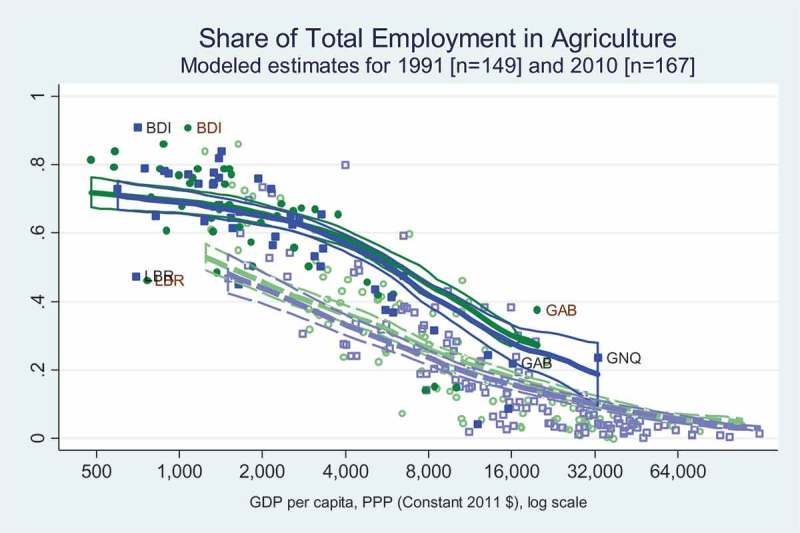
Agricultural employment is far higher in Africa than the rest of the world.

The data visualisation in Figure 3 combines both African and other countries in one panel, because the two regions’ employment shares are so different. The Preston Curve shows primary employment in agriculture to be consistently above 60 per cent in Africa’s poorer countries and around 30 per cent in Africa’s richest countries, whereas it ranges from below 50 per cent to under 20 per cent elsewhere at comparable income levels. This difference has not changed from 1991 to 2010, and is associated with the high rates of rural population growth shown in Figures 1 and 2 that reduce land area per household, holding back transformation and leaving more people in agriculture as shown in Figure 3.

#### 3.1.3. Labour productivity

Africa is distinctive not only because so many of its workers are in agriculture as shown above, but also because those agricultural workers have much lower productivity than nonfarm workers as shown below.

**Figure 4.**
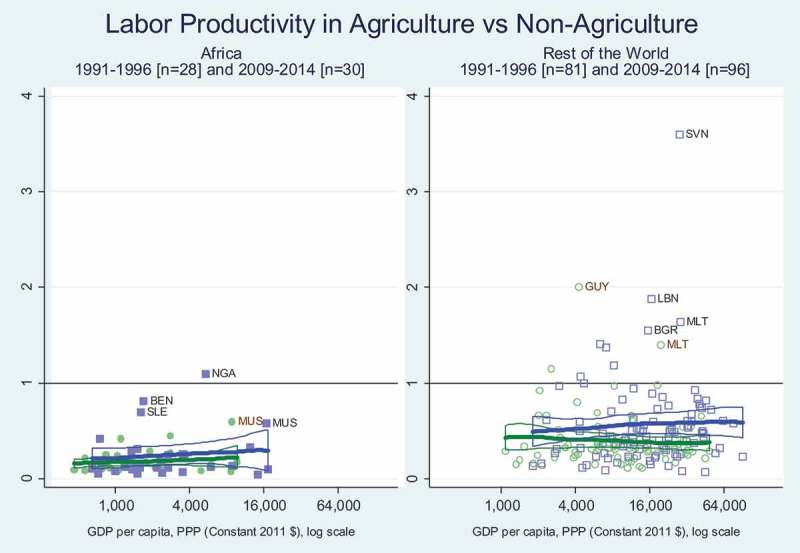
Africa’s labour productivity in agriculture relative to non-agriculture has not risen.

Figure 4 plots each country’s total agricultural value added per agricultural worker, divided by the country’s non-agricultural value added per non-agricultural worker. This uses the same ILO data source as Figure 3, showing the total value of all outputs produced by each sector (where agriculture includes forestry, hunting and fishing) minus the value of intermediate inputs used by each sector, divided by the number of workers in each sector. At each level of national income per capita, agriculture clearly offers much lower value added per worker than non-agriculture. The difference is accounted for partly by smaller quantities of capital and other factors per worker, and partly by lower productivity per unit of each factor, including labour.

A remarkable feature of Figure 4 is the statistically significant rise from 1991 and 2010 in relative productivity for agricultural labour outside of Africa but only at higher income levels. In Africa, there is no significant shift from the earlier to the later regression lines, and no significant upward slope associated with higher incomes. In other words, while the richer parts of the rest of the world experienced an upward shift in agricultural labour productivity relative to non-agricultural labour productivity, African farmers did not benefit from this increase in resources and productivity per agricultural worker.

The difference in productivity between agriculture and non-agriculture shown in the ILO data on Figure 4 is not merely an artefact of data collection methods. Gollin, Lagakos, and Waugh (2014) control for various other factors and confirm that Africa’s agricultural workers have significantly lower productivity than non-agricultural workers. This is consistent with Timmer (2009) and many other assessments, reflecting Africa’s delayed demographic and structural transformations that leaves rising numbers of African workers no choice but to try to feed themselves using limited resources with a declining area of land per worker.

### 3.2. Nutrition transition

The agricultural transformation described above involves a gradual transition from primarily rural, mainly agricultural employment at relatively low incomes to primarily urban, mainly non-agricultural work at higher incomes. This change involves a corresponding transition in nutritional outcomes, which we analyse here in terms of child stunting, adult obesity, and then intake of healthy as opposed to unhealthy diets.

#### 3.2.1. Stunting

The most widely used measure of undernutrition in public health research is stunting, defined as the proportion of a population’s children with a height-for-age z scores (HAZ) less than two standard deviations below the median of a healthy population. The onset of stunting typically occurs in early childhood and persists thereafter, so that prevalence rates among children under five years of age provides a useful measure of malnutrition experienced in recent years. Here we report national averages from the authoritative UNICEF/WHO/World Bank joint database of anthropometric surveys (WHO, 2010).

**Figure 5.**
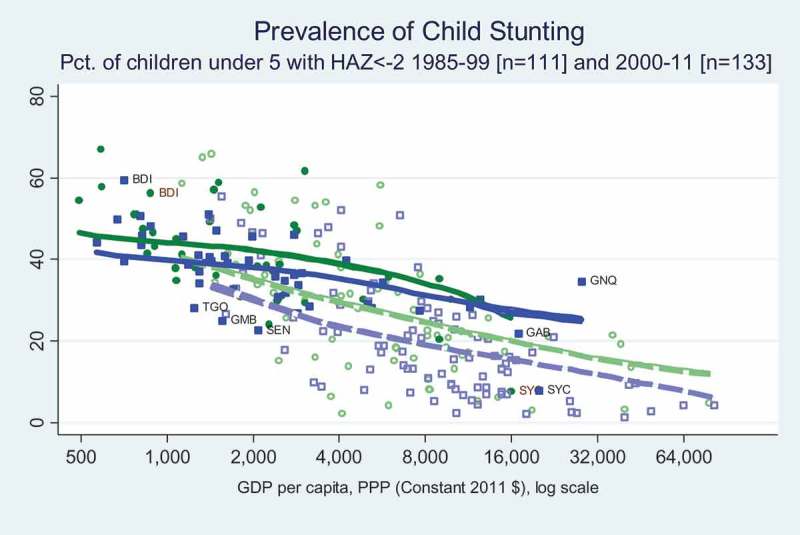
Child stunting rates have fallen sharply at all income levels.

Figure 5 reveals that Africa’s stunting rates have improved sharply over time at each income level, when comparing the earliest surveys in 1985–1999 to the most recent ones in 2000–2011. Africa differs from the rest of the world primarily in having a less steep income gradient, and correspondingly higher stunting rates at middle income levels. The few African countries we observe at those levels of per capita income have not been able to reduce child stunting in the way that countries elsewhere have done, perhaps because of their rapid rural population growth and low agricultural productivity.

#### 3.2.2. Obesity

Whether or not a person’s height was stunted in early childhood, lifelong weight gain through fat deposition is associated with risk for diabetes, cardiovascular disease and other disorders. A common threshold for high risk is obesity, defined in terms of Body Mass Index (BMI, in kg/m2) of 30 or more. For comparability across countries, we use modelled estimates of obesity prevalence in adults aged 20 or older, computed for 162 countries from the Global Burden of Disease 2013 study (Imamura et al., 2015).

**Figure 6.**
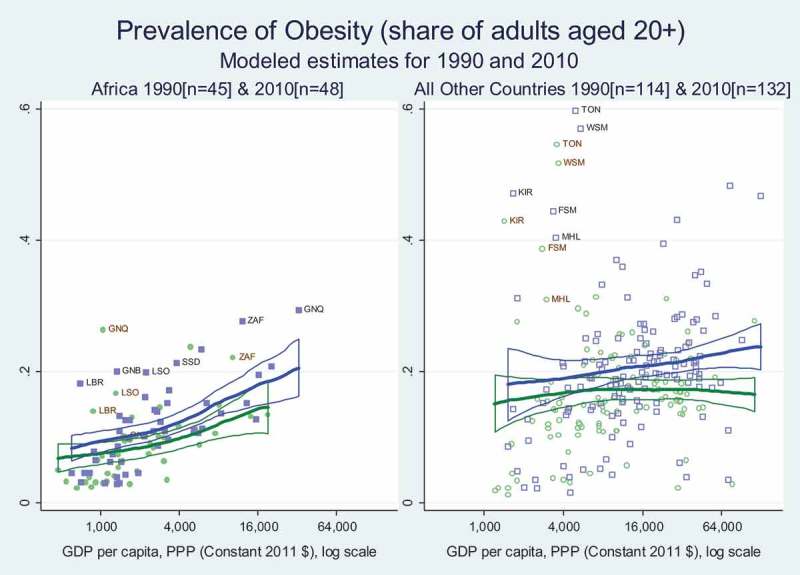
Adult obesity rates rise with income in Africa, and are below global averages.

Figure 6 reveals that Africa has a steep income gradient in adult obesity rates, but no upward shift from 1990 to 2010 at any income level. In contrast, for the rest of the world, there is a statistically significant upward shift from 1990 to 2010 for countries with per capita income levels exceeding about $16,000. Below that level there is relatively little income gradient in the rest of the world, and poorer African countries have significantly lower obesity rates than other countries at comparable income levels.

A notable aspect of these obesity data is their wide variability, as some African countries such as South Africa and oil-rich Equatorial Guinea had obesity rates of 22 per cent and 26.3 per cent, respectively. Conversely, the majority of low income African countries with per capita income below $4000 had very low adult obesity levels (<10%). A succession of reports such as FAO (2006), Abubakari, Lauder, Charles, and Bhopal (2008), Arojo and Osungbade  (2013) and NCD-RiskC (2016) have focused attention on increases in adult obesity in particular regions, which does pose a high risk for chronic disease, calling for sharp increases in detection and treatment of conditions such as diabetes and hypertension which have had low prevalence in the past but are and will continue to account for a rising share of Africa’s disease burden.

#### 3.2.3. Diet quality

Stunting and obesity are the most visible forms of malnutrition, but food quality can contribute to diet-related disease in people of any height and weight. Here we use the global dietary data assembled by NutriCoDE for the Global Burden of Disease study, producing modelled estimates for all countries in 1990 and 2010 of two distinct diet-quality measures based on mean adult intake of 10 dietary items for which more intake is healthier, and seven items for which less intake is healthier. Both diet quality indexes count all of their food items equally, and are normalised for a 2000 calorie diet in an ordinal scale from one to 100 where higher values mean a healthier diet (Imamura et al., 2015).

**Figure 7.**
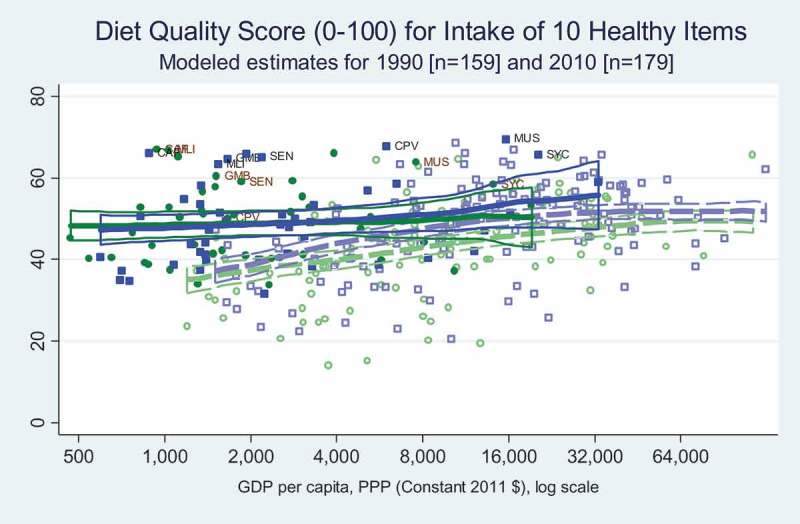
Diet quality over 10 healthy items is relatively good in Africa with no income gradient.

Figure 7 shows mean intake of 10 healthy dietary items at each level of income, revealing that African countries with per capita income below $4000 had significantly higher intake of these foods than others at similar income levels. Unlike Africa, the rest of the world has a strong income gradient with poor countries consuming low levels of these foods, converging up to higher levels observed in Africa over the income level of about $4000, with a small upwards shift from 1990 to 2010 in the rest of the world but not in Africa.

**Figure 8.**
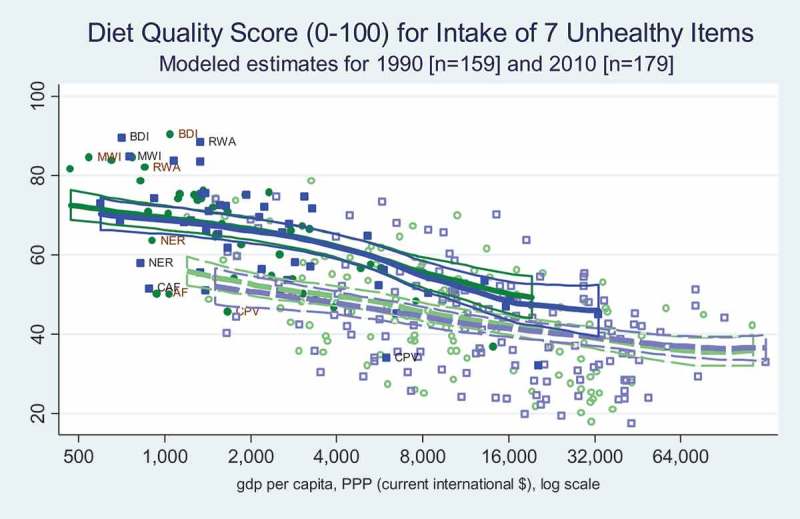
Diet quality over seven unhealthy items is better in Africa than elsewhere, at low incomes.

Figure 8 shows mean intake of seven unhealthy dietary components, expressed in a diet quality score so that less intake of these foods gives a higher score (closer to 100). Like the healthy-item score this reveals that African countries at most income levels have relatively better diet quality than the rest of the world at similar income levels, while diet qualities converge at higher income levels. Similar to the pattern observed through the healthy diets score, there was hardly any change in diet quality between 1990 and 2010 in African countries across all income levels.

#### 3.2.4. Food policy

**Figure 9.**
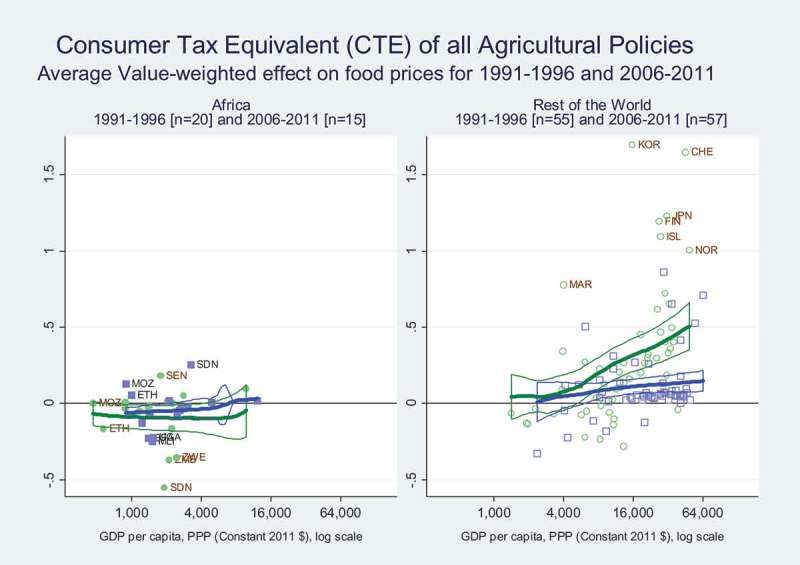
Food prices are kept low in Africa, and only somewhat increased elsewhere.

To measure cross-country differences in food policy we begin with Consumer Tax Equivalent (CTE) estimates from the World Bank’s Distortions to Agricultural Incentives project, using data originally compiled for Africa by Anderson and Masters (2009). The CTE is defined as the percentage change in the wholesale price paid for each food item, relative to the price that would have prevailed if government policy permitted free international trade and a competitive domestic market. CTEs are among the most direct and internationally comparable measure of food policy interventions, representing the tariff-equivalent percentage by which government policies raise or lower food prices. The data shown in Figure 9 are updated values from Anderson and Nelgen (2013), drawing on prices for major food products across 85 countries from 1950 to 2011. To focus on the cross-country pattern, we show the average CTE for all food items, weighted by their share of food spending, for the time periods 1991–1996 and 2006–2011.

Figure 9 reveals that African countries pursue policies that lower food prices, transferring real income from farmers to consumers, with no significant change in average CTEs over time and no gradient associated with national income. In contrast, observations from the rest of the world where incomes are higher show a steep income gradient in the 1991–1996 data which largely disappears in the 2006–2011 period. In the later period, governments outside Africa continued to raise food prices, although the price increase is smaller than it had been.

The 1991–1996 data across both panels to cover the world as a whole reveals the development paradox observed over much of the twentieth century, in which governments in poor countries reduce food prices while governments in richer countries do the reverse. This was considered paradoxical in that both kinds of policy favour a relatively richer minority at the expense of a poorer majority within each country. As shown in Figure 9, that paradox has largely though not entirely disappeared, with many African governments still taxing their agricultural producers to help food consumers, while other governments do much less taxing of food consumers to help farmers.

Beyond prices, another aspect of government policy is fiscal expenditure by sector. Here we present government spending on health (including nutrition) relative to agriculture (including fisheries), as compiled in the database of Statistics on Public Expenditures for Economic Development (SPEED) from the International Food Policy Research Institute (IFPRI, 2016). Collected from multiple sources, the SPEED dataset offers the best available global data on public expenditures for these two sectors.

**Figure 10.**
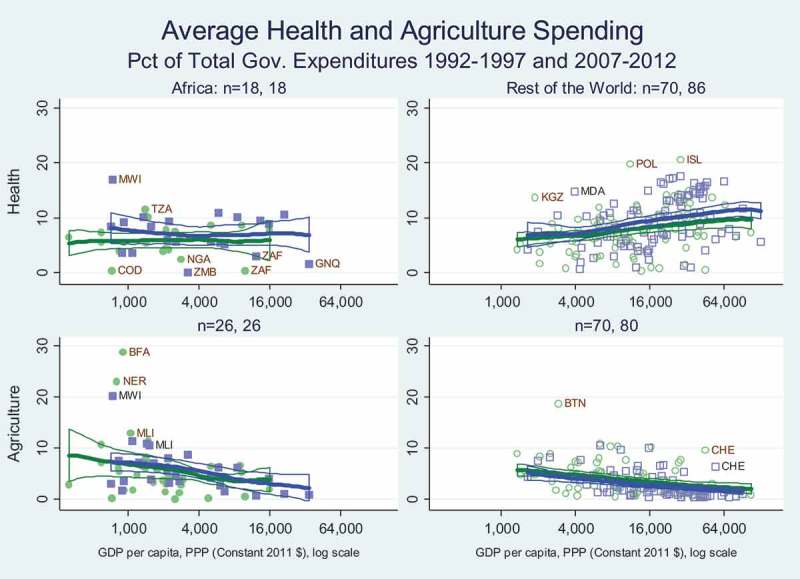
Government spending on agriculture and health have not shifted up.

Figure 10 shows public expenditures as percentages of total public spending, reflecting each government’s commitment to health and agriculture relative to other sectors. Spending levels for health along the top two panels are generally above those for agriculture along the bottom two panels, and spending on health shows an upward income gradient outside Africa while spending on agriculture has a downward income gradient in both Africa and elsewhere.

The SPEED data shown in Figure 10 support other reports that African governments are still investing far less in agriculture than their commitments under the 2003 Maputo and 2014 Malabo agreements (Mink, 2016). In these agreements they set targets of 10 per cent of government spending on agriculture, which only a very few countries reached in 2010.

## 4. Conclusions

This paper documents the timing and pattern of three broad changes associated with economic growth between the 1990s and the 2010s, comparing Africa with the rest of the world in terms of economic transformation from farm to nonfarm activity, nutrition transition from less stunting to rising obesity, and agricultural policy from lowering to raising food prices. Many factors contribute to the patterns we see. In this paper the aim is to describe stylised facts, notably about economic transformation in terms of rural population growth, agriculture’s share of total employment, and the productivity of agricultural workers relative to non-agricultural workers; about nutrition transition in terms of child stunting and adult obesity and diet quality with intake of healthy and unhealthy items; and about agricultural policy in terms of food prices and public investment levels.

For economic transformation, the main result we find is that Africa – compared to other regions, at each level of per-capita income – has faster rural population growth (Figures 1 and 2), a larger share of workers in agriculture (Figure 3) and lower labour productivity for them relative to non-agricultural workers (Figure 4). The pattern in rates of change observed across African countries are consistent with those observed elsewhere, but different in magnitude, with the central distinction being Africa’s relatively recent and rapid increase in the number of rural people and consequent decline in agricultural land, water and other rural resources per rural person. This pattern was present in the 1990s, remains present in the 2010s and is likely to persist even as African towns and cities grow faster than urban areas elsewhere, and as the share of African workers who are in agriculture declines.

The apparent paradox in economic transformation of year-to-year increases in the number of rural people who work in agriculture at low productivity, despite even faster growth in the number of urban people in nonfarm employment at higher productivity, is readily explained by Africa’s very high rate of total population growth and high initial share of workers in agriculture. As shown in our Figure 1, the central difference in economic transformation between Africa and the rest of the world concerns the timing and magnitude of total population growth, and the rural demographic momentum. This finding’s policy implication is that, to avoid continued impoverishment due to continued decline in agricultural land per rural person, African farmers would need to see increased public investment to increase land and labour productivity, as well as higher levels of public services and safety nets.

For nutrition transition, our main result is rapid improvement in child stunting at each level of income in both Africa and other regions (Figure 5). The downward shift in child stunting between the late twentieth century (1985–1999) and the early twenty-first century (2000–2011) reveals structural improvements in the determinants of child stunting. Our Preston Curve approach also reveals a strong income gradient, but primarily outside of Africa. Among Africa’s poorest countries stunting rates are the same as in other regions, but at higher national income levels there is much lower stunting elsewhere than in Africa. This finding is consistent with Africa’s demographic challenge described above, as the number of relatively low-productivity rural people continues to increase despite urbanisation and the rise of non-agricultural employment.

Improvements in child stunting contrasts sharply with trends in adult obesity (Figure 6), for which there has been no structural upward shift at each income level in Africa, although there is a strong income gradient among African countries and a significant upward structural shift among higher income countries in other regions. Africa’s relatively low adult obesity rates are consistent with higher quality diets as measured by the NutriCoDE indexes, showing that most African countries have more consumption of healthy dietary components (Figure 7) and less consumption of unhealthy items (Figure 8) than countries elsewhere at corresponding levels of income. But, income gradients differ between regions, and these diet quality indexes converge to similar levels in the highest income countries.

In food policy, our main result is that African governments continue to keep consumer prices of major farm products relatively low, benefiting consumers at the expense of producers (Figure 9). Governments elsewhere in higher-income countries continue to keep consumer prices relatively high, but with a large structural shift downwards in the income gradient as richer countries outside Africa did so to a much smaller degree in the recent 2006–2011 data than they did in the earlier 1991–1996 period. The longstanding development paradox is now much smaller in magnitude than it was during the twentieth century. Policy-makers in both Africa and elsewhere continue to use food price policy to tax their poorer citizens and provide concentrated benefits to more influential groups, who tend to be food consumers in Africa and other low-income countries but food producers in higher-income countries elsewhere.

Public investments in agriculture and health, including nutrition, have contrasting income gradients (Figure 10). The poorest African countries have similar levels of government spending across the two sectors, but there is a negative income gradient for agricultural expenditure so higher-income African countries spend less on agriculture than on health. The rest of the world also has a negative income growth for agricultural expenditure, whereas there is a positive gradient for health spending, so expenditure levels are higher for health, especially in the richest countries.

The Preston Curve results presented here focus on regional averages at each level of national income, using confidence intervals and scatter plots to see variation among countries within Africa and in the rest of the world. Beyond these national averages, further research addresses within-country variation in the kind of data shown here, and also extends these results to other kinds of data. This particular paper summarises only a few of the major challenges ahead for African agriculture, nutrition and food policy. We find that even if economic development proceeds rapidly in urban Africa, rural Africans will face continued impoverishment unless government interventions change to improve the lives of people working in agriculture at low productivity levels. Clear gains in child stunting reveal the potential for success despite these challenges, and could inspire interventions to achieve similar improvements in other aspects of African food systems.
